# Applications of Click Chemistry in the Development of HIV Protease Inhibitors

**DOI:** 10.1155/2018/2946730

**Published:** 2018-07-19

**Authors:** Mukesh M. Mudgal, Nagaraju Birudukota, Mayur A. Doke

**Affiliations:** ^1^Department of Chemistry and Biochemistry, Florida International University, 11200 SW 8th Street, Miami, FL 33199, USA; ^2^Department of Environmental and Occupational Health, Florida International University, 11200 SW 8th Street, Miami, FL 33199, USA

## Abstract

Acquired Immunodeficiency Syndrome (AIDS) has been devastating for millions of people around the world. Inhibition of the human immunodeficiency virus (HIV) protease is among the most important approaches for the therapeutic intervention in HIV infection. Since the discovery of the HIV-1 protease, this enzyme has been considered as a key target for the inhibition of viral replication. A large body of research has been done to develop an effective HIV-1 protease inhibitor. There are to date 10 HIV-1 protease inhibitor drugs approved by the Food and Drug Administration (FDA) that have improved the survival and quality of life of HIV infected people. These drugs are prescribed in combination with the reverse transcriptase inhibitors, which is referred to as highly active antiretroviral therapy (HAART). The HIV-1 protease inhibitors play a vital role in HAART. The applications of click chemistry are dispersing in the field of drug discovery. Recently, click chemistry has captured a lot of attention and has become a powerful tool for the synthesis of medicinal skeletons in the discovery of anti-HIV drugs. Click reaction is a well-known method for making carbon−heteroatom−carbon bonds. Click reactions are popular because they are wide in scope, of high yielding, quick to perform, and easy to purify. In this review, we outlined current approaches towards the development of HIV-1 protease inhibitors employing click chemistry.

## 1. Introduction

The first case of AIDS was reported in the United States in June 1981. AIDS became an epidemic that affected millions of people around the world [1]. According to a WHO report, 36.7 million people worldwide were living with HIV in 2016 [2]. Since the start of the epidemic, around 76.1 million people have become infected with HIV, 35.0 million of whom died. The Joint United Nations Programme on HIV/AIDS (UNAIDS) is determined to end the public health threat of the global HIV epidemic by 2030 [3]. To achieve this ambition, an estimated budget of US$ 26.2 billion will be required for the HIV response in 2020, which may gradually reduce to $22.3 billion by 2030 [4].

HIV is a retrovirus that if left untreated can lead to AIDS [5]. HIV spreads through certain body fluids and attacks the immune system, the CD4 cells (T cells) in particular, which assist the immune system in fighting off infections. Gradually, HIV can destroy the immune system and the body cannot fight off infections and diseases. The life cycle of HIV starts with the entry of free virus into the bloodstream. The free virus circulates in the bloodstream, attaches itself to the surface of a cell, and discharges its contents into the host cells. The enzyme reverse transcriptase uses the genetic material, ribonucleic acid (RNA) of the HIV, to build the HIV deoxyribonucleic acid (DNA). The HIV DNA gets inserted into the host's chromosome with the assistance of an enzyme, HIV integrase, and this establishes the HIV infection in the cell. The activated HIV DNA makes the raw material for new HIV viruses. The immature virus releases out from the infected cell and in this process of maturation, protease enzymes cut the raw materials and assemble into a functioning virus [6].

**Figure 1:**
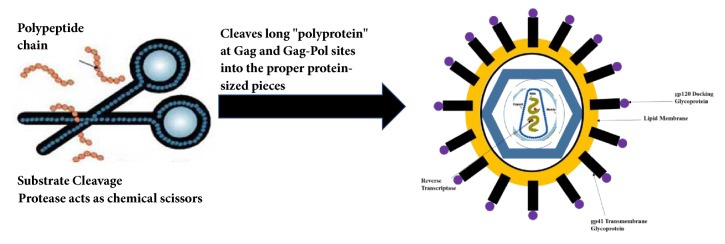
HIV protease as chemical scissors.

Antiretroviral therapies (ART) are medications that do not kill the HIV virus but can prevent the growth of the virus. When the growth of the HIV virus is slowed down, the HIV disease progression also slows down. There are six main types of antiretroviral drugs that currently exist based on the stage in the viral life cycle where they are targeted: (i) nucleoside reverse transcriptase inhibitors (NRTIs) and nucleotide reverse transcriptase inhibitors (NtRTIs), that work by blocking the reverse transcriptase enzyme so that HIV cannot make new virus copies of itself; (ii) non-nucleoside reverse transcriptase inhibitors (NNRTIs), which work by blocking the enzyme reverse transcriptase and prevent reverse transcription, thus stopping HIV replication; (iii) protease inhibitors (PIs), that work by blocking the activity of protease enzymes. HIV uses enzyme protease to break up large polyproteins into smaller pieces, which are required for the assembly of new viral particles (Figure 1).

**Figure 2:**
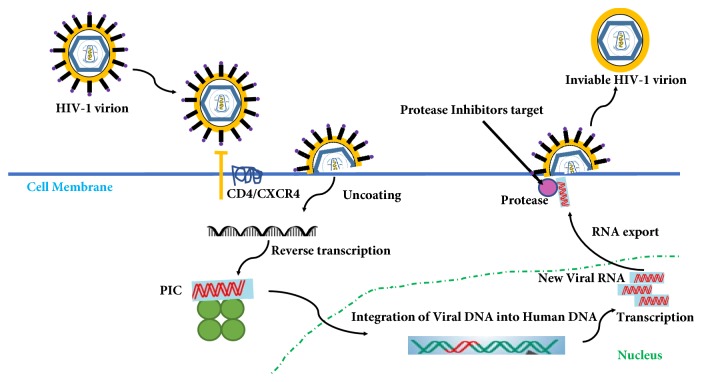
Schematic of HIV protease inhibition.

The HIV can still replicate but the resulting virions are immature and cannot infect new cells (Figure 2); (iv) fusion inhibitors act by blocking the fusion of HIV envelope with the host CD4 cell membrane, thus preventing the entry of HIV into the CD4 cells; (v) chemokine receptor antagonists (CCR5 Antagonists) act by blocking the CCR5 coreceptor and prevent HIV from entering the cell; (vi) integrase inhibitors work by stopping the virus from integrating with DNA of human cells.

Combination of the antiretroviral drugs is referred to as HAART, considered as the most effective treatment model for AIDS. Protease inhibitors play a very important role in HAART [7]. After the advent of AIDS-related HAART treatment, mortality has dropped sharply, and AIDS has become a controllable disease. Currently, there are 33 FDA approved drugs for the treatment of HIV infection, 10 of which are HIV protease inhibitors. Drug-resistant and cross-resistant mutant HIV-1 PRs have been identified. However, protease inhibitor-based therapy has a lower level of resistance compared to other classes of antiretroviral therapies like non-nucleoside reverse transcriptase inhibitor- (NNRTI-) based therapy [8]. Poor bioavailability and toxicity are the common disadvantages of protease inhibitor-based therapy, although the toxic effects could result from the drug-drug interaction and overdose. Thus, the discovery of new, safe, and potent protease inhibitors that are less prone to the development of resistance is urgently needed.

## 2. HIV-1 Protease

### 2.1. Structure of HIV-1 Protease

Drugs that are designed to attack HIV-1 protease are among the stellar breakthroughs of modern medicine. HIV protease's protein structure was first reported in 1989 and later has been deeply studied and investigated using X-ray crystallography. The studies of X-ray crystallography revealed the structure of HIV-1 protease exists as a homodimer [9]. HIV-1 protease consists of identical subunits, each made up of 99 amino acids. The active site lies between the identical subunits and has the characteristic Asp-Thr-Gly (Asp25, Thr26, and Gly27) sequence common to aspartic proteases. The two-subunit chains assemble to form a long tunnel covered by two flexible protein “flaps” which move a distance of up to 7 Å when the enzyme becomes associated with a substrate [10]. A water molecule acts as a nucleophile along with aspartic acid to hydrolyze the scissile peptide bond present at the center of the tunnel. This process is used to break the protein chain [11]. It is impossible to decipher the structure of the active form of HIV-1 protease as the chain would be cleaved before the structure could be solved [12].

### 2.2. Function of HIV-1 Protease

The advancement of crystallographic techniques improved the understanding of HIV-1 protease structure and made it the most well-studied enzyme known to medicine. The HIV-1 protease is a retroviral aspartyl protease that is essential for the life cycle of HIV, the retrovirus that causes AIDS [13]. HIV protease plays an important role in the cleavage of long polyprotein at Gag and Gag-Pol sites into the proper protein-sized pieces to create the mature protein components of an infectious HIV virion [14]. Inhibition of HIV protease activity can cause improper cleavage of protein components which results in an inviable and ineffective HIV virion [15]. Thus, inhibition of HIV protease activity disrupts HIV's ability to replicate and infect, which makes it a target of choice for researchers [16, 17].

## 3. Click Chemistry

**Figure 3:**
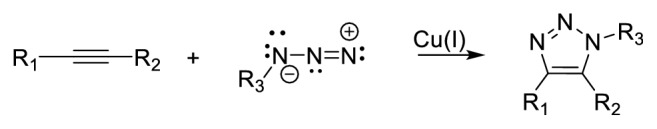
Huisgen [3 + 2] cycloaddition reaction: the copper-catalyzed 1,3-dipolar cycloaddition of an azide to an alkyne to create 1,2,3-triazoles.

Recently, click chemistry has emerged as a powerful tool in drug discovery to synthesize compounds containing carbon-heteroatom-carbon bonds [18, 19]. Click chemistry is a chemical approach that employs the copper (I) catalyzed 1,2,3-triazole formation from azides and terminal acetylenes to yield useful and versatile compounds (Figure 3). Triazole is an interesting class of compounds which exists in two types, the 1,2,3-triazoles and the 1,2,4-triazoles. The synthesis and biological applications of 1,2,3-triazoles and 1,2,4-triazoles have previously been extensively studied [20–23]. The “click chemistry reaction”, defined by noble laureate KB Sharpless and associates in 2001 [24, 25], served as a model reaction for the generation of novel pharmacophores.

The click reactions are popular because click chemistry employs chemical reactions that are wide in scope and of high yielding and produce very little byproduct that can be removed easily by chromatographic techniques. These reactions are quick and easy to perform and are stereospecific. Carbon-carbon double bond or carbon-carbon triple bond provide energy for click connection and therefore serve as a starting material for click reactions. Click chemistry is the powerful reaction for making carbon−heteroatom−carbon bonds (mostly N, O, and S) in an aqueous environment [26, 27]. In this review, we would like to provide an overview of the application of click chemistry in the development of HIV protease inhibitors.

## 4. Recent Progress towards the Development of HIV-1 Protease Inhibitors

### 4.1. First Generation of Protease Inhibitors

**Figure 4:**
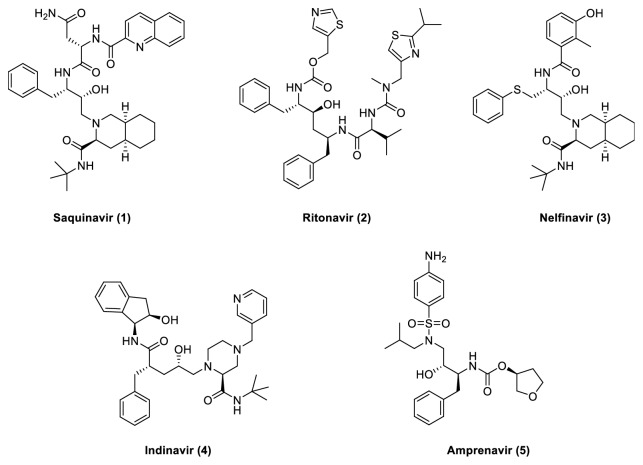
First-generation protease inhibitors.

Protease inhibitors were designed to mimic the transition state of the protease's actual substrates. A peptide linkage consisting of –NH-CO- is replaced by a hydroxy ethylene group (-CH2-CH(OH)-) which makes it difficult for the protease to cleave the linkage. HIV protease inhibitors fit the active site of the HIV aspartic protease given the aspartyl protease's mode of action. The most promising transition state mimic was hydroxyethylamine, which led to the discovery of the first protease inhibitor, Saquinavir (**1**) (Figure 4).

Ritonavir (**2**), a peptidomimetic HIV protease inhibitor, was designed to fit the C2-symmetry in the binding site of the protease, while Nelfinavir (**3**) was the first protease inhibitor that was not peptidomimetic. It was designed as a nonpeptidic inhibitor; an iterative protein cocrystal structure of the inhibitors was replaced by nonpeptidic substituents [28].

### 4.2. Second-Generation Protease Inhibitors

**Figure 5:**
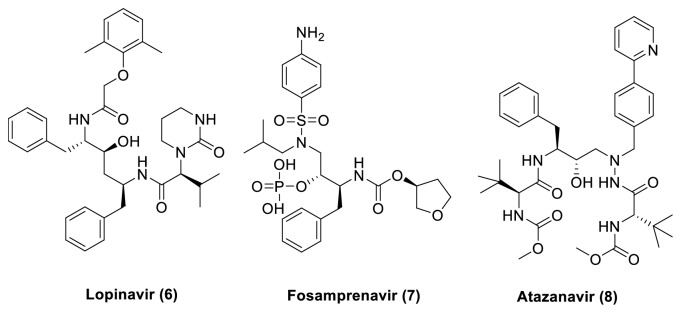
Second-generation protease inhibitors.

The first generation of HIV protease inhibitors, including Saquinavir (**1**), Ritonavir (**2**) Nelfinavir (**3**), Indinavir (**4**), and Amprenavir (**5**) (Figure 4), was initially effective against HIV infection; the fast-emerging resistance to these agents has been a substantial and persistent problem in the treatment of AIDS. Mutations in and outside of the active site of the HIV protease enzyme, including those at protease cleavage sites in the Gag-Pol polyprotein precursors that code for alterations of the conformational shape, facilitate resistance of HIV to protease inhibitors [29]. This led to the development of new therapies to cure AIDS focused on avoiding cross-resistance to drugs that are already on the market [13]. A new second generation of protease inhibitors (PI), Lopinavir (**6**), Fosamprenavir (**7**), and Atazanavir (**8**) (Figure 5), has potent activity against HIV.

### 4.3. HIV-1 Protease Inhibition Using* In Situ* Click Chemistry

Click chemistry, as explained by K. Barry Sharpless, is the use of molecular building blocks with built-in high-energy contents that are designed to link together selectively and covalently [24]. The* in situ* click chemistry is the expansion of the click chemistry strategy, where the chemical and biological receptor structures serve as templates to direct the formation of click chemistry products. The term small molecule* in situ* click chemistry (SISC) was coined by Sharpless and coworkers in 2002 [30]. They designed the small molecule inhibitors of acetylcholinesterase. The principle behind the methodology is that the 1,3-dipolar Huisgen cycloaddition reaction can be catalyzed without a metal catalyst provided that the two reactants are brought in close proximity to each other in proper orientation by the protein target.

**Figure 6:**
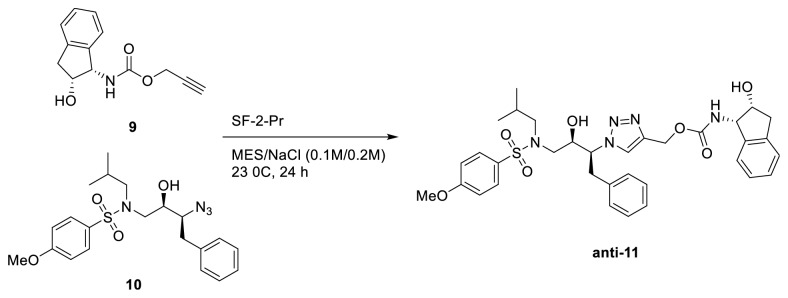
*In situ* click chemistry reaction for the formation of protease inhibitor*** anti-11***.

In 2006, Whiting* et al*. reported a role of* in situ* click chemistry to explain bio-orthogonal 1,3-dipolar cycloaddition of azide and alkynes in HIV-1-Pr inhibition [31]. The protease itself served as a template for the* in situ* click reaction in the formation of protease inhibitor*** anti-*11**, 1,4-triazole product and catalyzed its formation (Figure 6). The objective of the study was to discover novel compounds by engaging the biological target itself in the covalent assembly and selection of its inhibitors. The protease reaction was studied by incubating alkyne (**9**) and azide (**10**) in the presence of the HIV-1-Pr SF2-WTQ7K-Pr in a 2-morpholinoethanesulfonic acid buffer solution at 23°C for 24 h. Background reaction was performed in the absence of the enzyme. The comparison of HPLC/mass spectrometry analysis of both enzyme and background reactions revealed the tenfold increase of triazole*** anti-*11**, an inhibitor of the wild-type HIV-1-Pr (IC_50_ = 6nM,* K*_*i *_= 1.7nM) product with a much enhanced regioisomeric ratio in the presence of the enzyme. Another experiment was conducted by adding the inhibitor HIV-1-Pr active site ligand (TL3) in background reaction which showed no effect and suppressed the product formation in the presence of an enzyme. This demonstrates that the active site of a biological target is involved in the cycloaddition reactions.

### 4.4. Click Chemistry in Rapid* In Situ* Screening of HIV Protease Inhibitors

**Figure 7:**
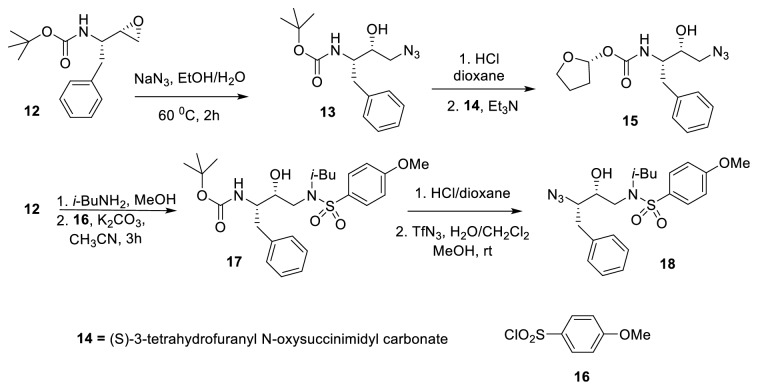
Synthesis of azide cores** 15** and** 18**.

Click chemistry has emerged as a robust method for the microscale synthesis and* in situ* biological screening of molecules. It guarantees the high yield and purity of the desired products without isolation of the product and manipulation of the functional groups. In 2003, Brick* et al. *reported synthesis of HIV-1 PR inhibitors in the microtiter plate format to generate the desired libraries of inhibitors and subsequently screened them in microtiter itself against HIV-1 PR and its mutants without any purification and isolation [32]. The synthesis of novel HIV-1 PR inhibitors was achieved by employing Cu(I) catalyzed click chemistry approach. The importance of hydroxyethylamine moiety has been studied extensively as a backbone replacement of amide bonds in the P1/P1' position of aspartyl protease inhibitors like Amprenavir [33], Nelfinavir [34], and Saquinavir [35]. The synthesis of novel HIV-1 PR inhibitors was achieved by diversifying the P2/P2' while retaining the hydroxyethylamine core moieties. Two different azide core molecules were synthesized from optically active epoxy amine (**12**) (Figure 7). The opening of epoxide with azide, acid catalyzed removal of the Boc group followed by free amine coupling with carbonate group provided first azide core molecule (**15**). The synthesis of the second azide molecule (**18**) was achieved through employing the same epoxy amine (**12**), by the opening of epoxide with isobutylamine and subsequent coupling of the generated secondary amine with* p*-methoxybenzenesulfonyl chloride. The desired product (**18**) was obtained by eliminating the Boc group followed by diazo transfer reactions.

**Figure 8:**
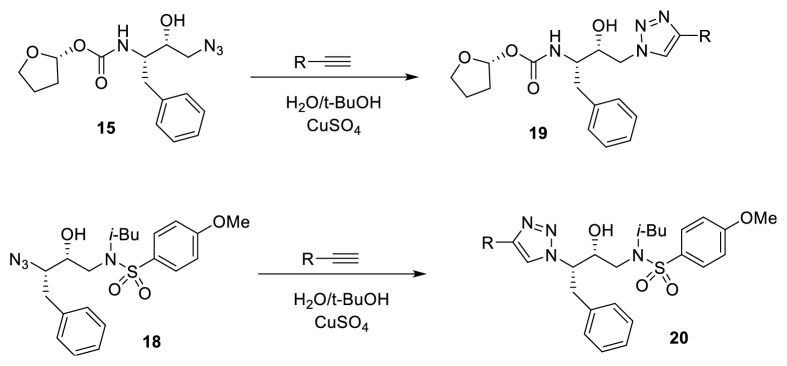
Click chemistry reaction to generate a library of HIV PR inhibitors.

Each of these azide compounds was subject to reaction with fifty different alkyne moieties by using the CuAAC click chemistry approach. After incubating the reaction mixture for 48h at RT, LC-MS analysis confirmed the formation of a quantitative amount of corresponding cycloaddition products (**19 **and** 20**) (Figure 8).

**Figure 9:**
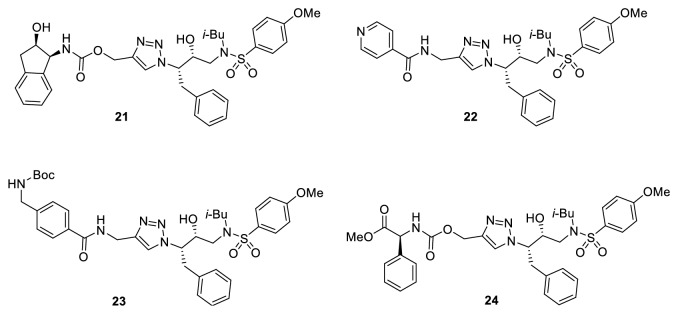
Promising HIV-1 PR inhibitors.

Four of these synthesized compounds (**21-24)** (Figure 9) derived from the azide** 18 **showed good activity against the HIV-1 PR and three mutants (G48V, V82F, and V82A).

### 4.5. Click Chemistry in the Synthesis of Coumarin Derivatives as Dual Action HIV-1 Protease and Reverse Transcriptase Inhibitors

**Figure 10:**
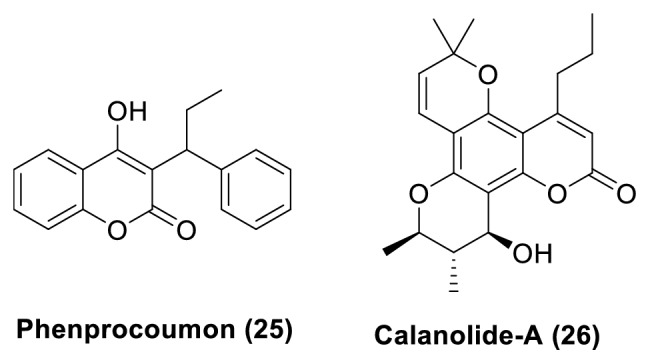
Phenprocoumon** (25)**, a non-peptidic HIV-1 PR inhibitor, and Calanolide-A** (26)**, a non-nucleoside HIV-1 RT inhibitor.

Coumarin derivatives are known to exhibit anticancer, anti-HIV, antifungal, and antibacterial activities. Phenprocoumon (**25**), a non-peptidic HIV-1 PR inhibitor, and Calanolide-A (**26**) (Figure 10), a non-nucleoside HIV-1 RT inhibitor, are the leading examples in this class.

**Figure 11:**
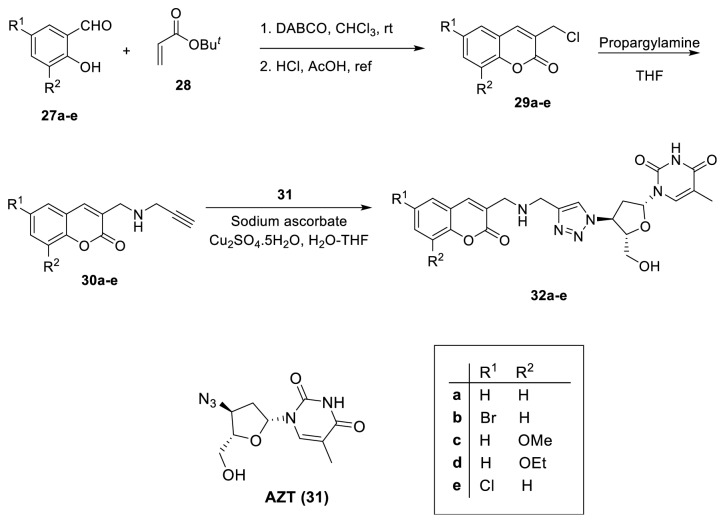
Synthesis of triazole derivatives** 32a-e**.

In 2010, Olomola* et al. *reported the synthesis of a series of compounds (**32a-e**) containing both coumarin and triazolothymidine (AZT-**31**, a well-known HIV-1 RT inhibitor) moieties to obtain dual action HIV-1 PR and RT inhibitions (Figure 11) [36]. Synthesis of alkynylated coumarin derivatives (**30a-e**) was achieved by employing the DABCO-catalyzed Baylis-Hillman reaction of salicylaldehydes (**27a-e**) and* t-*butyl acrylate (**28**), to afford intermediate adducts, acid catalyzed cyclisation of which afforded 3-(chloromethyl)coumarins (**29a-e**). The nucleophilic substitution attack of nitrogen nucleophile of propargylamine on the exocyclic methylene center provided corresponding substitution products (**30a-e**). Subsequently, employing the Cu (I) catalyzed click chemistry, the alkynylated coumarin intermediates were subjected to cycloaddition with the azidothymidine compound (**31**) to obtain 1,2,3-triazole moieties** (32a-e)**.

**Figure 12:**
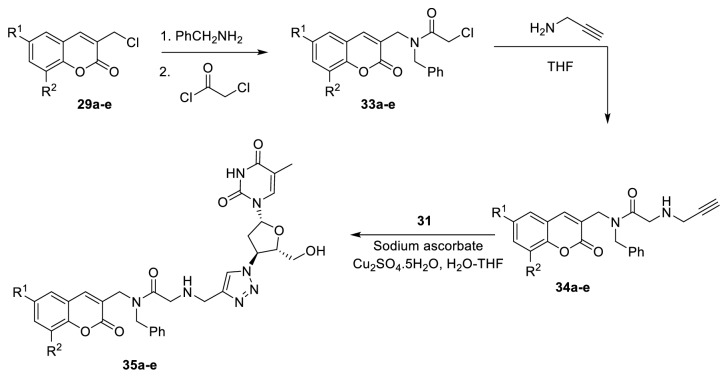
Synthesis of coumarin-AZT conjugates.

The ethylene dipeptide HIV-1 PR inhibitors usually contain benzyl groups. Therefore, Olomola* et al. *in 2013 reported the synthesis of another series of* N-*benzylated amido analog of coumarin-AZT conjugates (**35a-e**) with a similar chemical sequence (Figure 12) [37]. The* N*-benzylated amido group was introduced by treatment of the intermediates (**29a-e**) with benzylamine followed by treatment with chloroacetyl chloride. Nucleophilic substitution reaction of propargylamine with chloroacetamide (**33a-e**) afforded alkynylated products (**34a-e**), which upon subjection to Cu (I), catalyzed click chemistry with AZT, afforded coumarin-AZT (**31**) conjugates (**35a-e**).

Saturation transfer difference (STD) NMR, enzyme-inhibition, and computer modeling techniques have been employed to evaluate the potential of the* N*-benzylated cycloaddition products (**35a-e**) and a series of non-benzylated analogs (**32a-e**) as a twofold action of HIV-1 PR/RT inhibitor.

## 5. Conclusion

This review focuses on the application of click chemistry in the development of HIV-1 protease inhibitors. The applications of click chemistry are spreading, ranging from discovery through combinatorial chemistry to the field of proteomics employing the bioconjugation reactions. Click chemistry played a vital role in the antiviral drug discovery, and it can be anticipated that in the future it will make further contributions in anti-HIV drug discovery. Scientists around the world have been trying to develop the medicinal drugs to combat HIV. HIV medicines will help people to live longer, healthier lives and also reduce the risk of transmission and control the spread of disease. The HIV protease inhibitors continue to play a very important role in HAART. There are some side effects associated with the antiretroviral drugs, depending on the individual body type and the level of HIV infection. Therefore, drug therapies that are more efficient have fewer side effects and drug-related toxicities are required. This critical review summarizes the progress made so far in the discovery of HIV-1 protease inhibitors employing innovative approaches. The click chemistry-based approach has become a popular protocol to synthesize HIV-1 protease inhibitors. A lot of research is still needed in the area of antiretroviral drug development to discover new antiretroviral molecules with improved potency that will reduce side effects and provide a new mechanism of actions.
